# Differential Requirement of *Gata2a* and *Gata2b* for Primitive and Definitive Myeloid Development in Zebrafish

**DOI:** 10.3389/fcell.2021.708113

**Published:** 2021-09-13

**Authors:** Oscar A. Peña, Alexandra Lubin, Jasmine Rowell, Yvette Hoade, Noreen Khokhar, Hanna Lemmik, Christopher Mahony, Phoebe Dace, Chianna Umamahesan, Elspeth M. Payne

**Affiliations:** ^1^Research Department of Haematology, Cancer Institute, University College London, London, United Kingdom; ^2^Department of Pathology and Immunology, School of Medicine, University of Geneva, Geneva, Switzerland; ^3^National Institute for Health Research (NIHR)/UCLH Clinical Research Facility, University College London Hospitals NHS Foundation Trust, London, United Kingdom

**Keywords:** *Gata2a*, *Gata2b*, hematopoiesis, stem cell, myelopoiesis

## Abstract

Germline loss or mutation of one copy of the transcription factor GATA2 in humans leads to a range of clinical phenotypes affecting hematopoietic, lymphatic and vascular systems. GATA2 heterozygous mice show only a limited repertoire of the features observed in humans. Zebrafish have two copies of the Gata2 gene as a result of an additional round of ancestral whole genome duplication. These genes, Gata2a and Gata2b, show distinct but overlapping expression patterns, and between them, highlight a significantly broader range of the phenotypes observed in GATA2 deficient syndromes, than each one alone. In this manuscript, we use mutants for Gata2a and Gata2b to interrogate the effects on hematopoiesis of these two ohnologs, alone and in combination, during development in order to further define the role of GATA2 in developmental hematopoiesis. We define unique roles for each ohnolog at different stages of developmental myelopoiesis and for the emergence of hematopoietic stem and progenitor cells. These effects are not additive in the haploinsufficient state suggesting a redundancy between these two genes in hematopoietic stem and progenitor cells. Rescue studies additionally support that Gata2b can compensate for the effects of Gata2a loss. Finally we show that adults with loss of combined heterozygosity show defects in the myeloid compartment consistent with GATA2 loss in humans. These results build on existing knowledge from other models of GATA2 deficiency and refine our understanding of the early developmental effects of GATA2. In addition, these studies shed light on the complexity and potential structure-function relationships as well as sub-functionalization of Gata2 genes in the zebrafish model.

## Introduction

GATA2 is a zinc finger transcription factor expressed widely across a number of tissues including vascular, lymphatic, urogenital, cardiovascular, neural, and hematopoietic systems. It is a master regulator of hematopoiesis with the majority of knockout mice succumbing to death from anemia before embryonic day E10.5 (Tsai et al., 1994). Its importance in human disease is also dominated by its promiscuous role in hematopoietic malignancies and immunodeficiencies. Inherited mutations in GATA2 cause a range of overlapping clinical syndromes; the monoMAC (monocytopenia with mycobacterium avium complex) syndrome, DCML (dendritic cell and myeloid leukemia), Emberger syndrome (lymphedema with predisposition to AML), Familial myelodysplastic syndrome (MDS), aplastic anemia and congenital neutropenia (Dickinson et al., 2011; Hahn et al., 2011; Hsu et al., 2011; Ostergaard et al., 2011; Pasquet et al., 2013). Mutations are found spanning the full length of the gene including missense, non-sense, indel, and regulatory region mutations, all resulting in functional haploinsufficiency. There are variable reports of genotype/phenotype correlations; lymphedema is reportedly more frequently associated with null mutations (Spinner et al., 2014), and missense mutations were more commonly associated with the progression to acute leukamia as reported in a review of French and Belgian cases (Donadieu et al., 2018). Somatic GATA2 mutations are also seen in hematopoietic malignancies with a strong association with acute myeloid leukemia (AML) with bi-allelic CEBPA mutations, or reduced expression of CEBPA such as in AML with t(8;21) (Greif et al., 2012; Christen et al., 2019). By contrast to inherited mutations, these somatic GATA2 mutations are clustered within the first zinc finger and phenotypically are enriched for erythro-leukemias (Ping et al., 2017). GATA2 also plays a role in other hematopoietic malignancies with the acquisition of neomorphic mutations in the second zinc finger of GATA2 resulting in poorer outcomes in patients with chronic myeloid leukemia (Zhang et al., 2008).

Germline mutations in GATA2 show phenotypes across all ages. However, are particularly enriched in children and teenagers presenting with myelodysplastic syndrome (MDS) and congenital neutropenia (Pasquet et al., 2013; Wlodarski et al., 2016). This highlights that critical early developmental requirements for GATA2 in hematopoietic specification and differentiation may predispose to the development of hematopoietic malignancies later in life.

The GATA family of transcription factors are conserved through evolution. Mammals have six GATA genes derived from two ancestral GATA genes identified in early deuterostome genomes as GATA123 and GATA456 orthologs (Gillis et al., 2009). The subsequent development of human GATA genes, GATA1-6, have evolved through two sequential rounds of whole genome duplication in vertebrates. Zebrafish have undergone a further round of genome duplication resulting in two ohnologs of the mammalian *GATA2* gene termed *gata2a* and *gata2b*. Both genes are highly homologous to the human GATA2 gene, particularly in the zinc finger domains where they are 98% and 95% identical at the amino acid level, respectively (Supplementary Figures 1A,B). Furthermore, the chromosomal regions surrounding zebrafish *gata2a* and *gata2b* show synteny with regions of human Chromosome 3 where GATA2 is located (Supplementary Figure 1C). Interestingly, while Gata2a has closer homology at the amino acid level to human GATA2, Gata2b is more closely syntenic with the GATA2 surrounding regions in humans. Prior studies have shown sub-functionalization of these genes in zebrafish with expression of Gata2b found only in branchiomotor neurons and hematopoietic stem and progenitor cells (HSPC) during development. Gata2a has a broader expression pattern reflecting that observed in mammals (Butko et al., 2015). Nonetheless, single cell sequencing studies of adult HSPC using transgenic Tg(*itga2b*:eGFP) zebrafish show that in adult hematopoiesis, both Gata2a and Gata2b are expressed in HSPC in an “or” fashion (Macaulay et al., 2016). This implies that both, or either, are required for HSPC function and that they have capacity to regulate expression of one another.

We set out to model GATA2 deficient human diseases in zebrafish by knocking out both Gata2a and Gata2b. We hypothesized that heterozygous loss of both genes would be required to faithfully model the human disease. Furthermore, because of the sequence and expression variability between the two ohnologs, we hypothesized that dissecting the role of loss of these genes alone or in combination would infer a degree of structure-function relationship of the GATA2 gene relevant to disease phenotypes.

Here, we demonstrate that Gata2a is required for normal primitive myeloid development and Gata2b is dispensable for this, while Gata2b orchestrates definitive developmental myelopoiesis. We show that Gata2a and Gata2b both contribute to the development of HSPC from the dorsal aorta but are not additive or synergistic in this manner in the heterozygous state. Finally, rescue studies suggest that Gata2b is able to partially complement Gata2a by rescuing defective vasculature and circulation, defining a mechanism whereby the cross-regulation of these genes in specific tissues may compensate for genetic loss.

## Materials and Methods

### Zebrafish Husbandry, Mutant, and Transgenic Strains

Wild-type, mutant and transgenic zebrafish (*Danio rerio*) stocks were maintained according to standard procedures in UK Home Office approved aquaria (Westerfield, 2007). Transgenic and mutant strains used in this study were *gata2a^*um*27^* carrying a 10 bp deletion in Gata2a (a gift from Nathan Lawson)*^7^, Tg(lyzC:nfsB-mCherry)* (a gift from Stephen Renshaw), *Tg(kdrl:GFP)^*la*116^*, *Tg(itga2b:GFP)^*la*2T*g*^* (Hsu et al., 2004; Lin et al., 2005; Choi et al., 2007; Zhu et al., 2011; Buchan et al., 2019).

Embryos were raised at 28.5°C in E3 medium (5 mM NaCl, 0.17 mM KCl, 0.33 mM CaCl_2_, 0.33 mM MgSO_4_) in Petri dishes. Pigment formation was avoided by supplementing E3 medium with phenylthiourea (PTU) (Sigma) 0.2 mM from 24 hpf or removed by bleaching post *in situ* hybridization by incubating in 5% v/v deionized formamide, 5% v/v H_2_O_2_, 0.5x SSC. For live experiments and prior to fixation, embryos were anesthetized with ethyl 3-aminobenzoate methanesulfonate (E10521, Sigma). Embryos and larvae were staged according to Kimmel et al. (1995), and larval ages are expressed in somite stage (ss), hours post-fertilization (hpf), and days post-fertilization (dpf). All procedures complied with UK Home Office guidelines.

### Generation of *Gata2b^*u5008*^* Mutant Line

We generated a zebrafish *gata2b* mutant line, *gata2b^*u*5008^* using CRISPR/Cas9. Briefly a sgRNA targeting exon 3 in *gata2b* gene was designed using CHOPCHOP v2^1^ (Labun et al., 2016) and guide synthesized according to (Gagnon et al., 2014) (see Table 1 for sequence). Cas9 mRNA was synthesized from the pT3TS-nCas9n plasmid, a gift from Wenbiao Chen (Addgene plasmid #46757) (Jao et al., 2013), using T3 mMESSAGE mMACHINE kit (AM1348, Ambion), following the manufacturer’s instructions. Cas9 mRNA and sgRNA were coinjected at the 1-cell stage at final concentrations of 380 and 65 ng/μL, respectively. *gata2b^*u*5008^* was identified by MiSeq of F1 progeny as carrying a 4 bp deletion ENSDARG00000009094.7:g.2011_2014del resulting in a truncating mutation, p.P172PfsX12 (Supplementary Figures 1A,D).

**TABLE 1:** Primers and guide sequences.

Primer name	Sequence
Kasp c- *gata2a*_*um27*	CAGAGGTAGTGGCCCGTTCCAT
Kasp x-*gata2a*_*um27*	GAAGGTGACCAAGTTCATGCTGTGGAGCCACCTC CACCC
Kasp y- *gata2a*_*um27*	GAAGGTCGGAGTCAACGGATTGTGGAGCCACCT CCACCG
Kasp c- *gata2b*_*u5008*	GGCCGGGTCCGGGGACACAT
Kasp x- *gata2b*_*u5008*	GAAGGTGACCAAGTTCATGCTTGCCTGCCGCCC ACCCCG
Kasp y- *gata2b*_*u5008*	GAAGGTCGGAGTCAACGGATTTGCCTGCCGCCC ACCCCA
*Gata2b* Op24 F	GATGCCGTGCCTGTCGATGA
*Gata2b* Op25 R	TTGTCGTTTGCGCCAGATGC
*Gata2b* Op44 F	TTTTTGCCAGCACGCCTTAC
*Gata2b* Op45 R	GGGTTGCTACGGATTGGACT
*Gata2a* um27 F	GGCCAGAACGTGGCTTTTCCG
*Gata2a* um27 R	GGGGATCACCAGGGGGTGAG
*Gata2b* sgRNA(PAM)	GGACACATCTTTGGGCGGGGTGG
*c-myb_probe_F*	CCAAGTCAGGAAAACGCCACCTCG
*c-myb_probe_R*	GCTGTTGTTTAGCGGAGTTGGGCT

### Whole Mount *in situ* Hybridization (WISH)

Whole mount *in situ* hybridization was performed as previously described (Thisse and Thisse, 2008). The *c-myb* WISH probe was generated by one-step PCR from 24 hpf mRNA using QIAGEN OneStep RT-PCR Kit using the primers in Table 1, and subsequently cloned into the pCRII TOPO vector. Other probes have been described elsewhere (Herbomel et al., 1999; Crowhurst et al., 2002; Kalev-Zylinska et al., 2002; Liu et al., 2007).

### Sudan Black and *o*-Dianisidine Stainings

Granulocytes in zebrafish were stained using Sudan Black (SB) as previously described (Le Guyader et al., 2008). SB positive (SB+) cells were quantified by manual counting from the distal end of the yolk extension to the tail tip in the caudal hematopoietic tissue (CHT).

*O*-dianisidine staining was used to label hemoglobinised erythrocytes in zebrafish larvae as previously described (Iuchi and Yamamoto, 1983).

### Analysis of Zebrafish by Flow Cytometry

Individual transgenic *Tg(itga2b:GFP)^*la*2T*g*^* larvae were analyzed by flow cytometry at 3 dpf as described previously (Payne et al., 2012).

### mRNA Rescue Experiments

Full length pCS2+:Gata2a was a gift from Barry Paw. The coding sequence of Gata2b was PCR amplified from embryo RNA using Superscript III Platinum one-step PCT kit (Invitrogen, Thermo Fisher Scientific), and TOPO cloned into pcDNA3.1V5-His. Forward orientation was confirmed by sequencing. mRNA was synthesized using Ambion mMessage mMachine (Ambion, Thermo Fisher Scientific) Sp6 or T7 ultra kit, respectively.

### Imaging and Image Processing

Fixed embryos and larvae were imaged either in 1x PBST or 80% v/v glycerol. Brightfield and epifluorescence images were acquired using a Leica M205 FA fluorescent dissecting stereoscope equipped with a Leica DFC 365 FX camera and LAS 4.0 software. Images were processed using Fiji version v1.49u software (Schindelin et al., 2012). Quantification of *c-myb* expression in Figure 2G and of *runx1* in Figures 3D, 4A were undertaken as described (Dobrzycki et al., 2018) with additional step of normalization to facilitate more accurate assessment between experiments. This was undertaken after background subtraction, by assigning the mean value of the WT for each independent experiment as 1 and normalizing all other values within the experiment to this. For image quantifications in Supplementary Figures 2D,H,L, images were pre-processed in Fiji and then segmented using ilastik version 1.3.3 (Berg et al., 2019). Pixel classification module was used for segmentation of images, and object classification module was used for selection of segmented objects. After quantification, relevant measurements for further statistical analysis were exported using a custom-written script in R, and background subtraction was carried out as previously described (Dobrzycki et al., 2018).

### Genotyping of *Gata2a^*um27*^* and *Gata2b^*u5008*^* Mutant Fish

Gata2a^um27^ fish were genotyped by PCR and restriction enzyme (RE) digest using *Msp*A1I, which is abolished by the 10 bp deletion, or Kompetitive allele specific PCR (KASP). G*ata2b^*u*5008^* genotyping was also conducted using PCR and RE digest with *Fau*I enzyme, which abolished by the deletion, or by KASP. Additional details can be located in Table 1.

### *In silico* Alignment and Synteny

GATA2 (human and zebrafish) protein sequences were aligned using Geneious^®^ software and similarities assessed using BLOSUM62 matrix. Synteny was assessed using the comparative genomics tools-region comparison on ENSEMBL for each GATA2 gene independently and subsequently for 10 genes upstream and downstream of each.

### Assessment of Adult Whole Kidney Marrow by Flow Cytometry

For these experiments a clutch of *Tg(itga2b:eGFP)*; *Gata2a^*um*27/+^*× *Tg(lyzC:mCherry);Gata2b^*u*5008/+^* embryos were raised in a single tank and genotyped just prior to analysis to mitigate for any tank specific effects. Adult zebrafish kidneys were dissected from terminally anaesthetized animals, dissociated in PBS with 1% FBS using a gentleMACs tissue dissociator (Milteyni) and analyzed by flow cytometry. Flow analysis was conducted using FlowJo^TM^.

### Statistical Analysis

Statistical analysis was performed using GraphPad Prism version 8.1.2 for OS X software (GraphPad Software, San Diego, CA, United States). The probability level for statistical significance was *p* < 0.05 and all tests used were two-tailed. Data are presented as mean ± standard deviation. Data from each independent experiment was normalized to the mean of its wildtype group, and experiments were pooled for analysis. No normality assumptions were made, and data was analyzed by Kruskal-Wallis test with Dunn’s multiple comparisons *post hoc* test or two-way ANOVA with Sidak’s *post hoc* test. In Figure 2Q, homozygote *gata2a^*um*27/um27^* embryos scored according to their circulation were tested for equal distribution with contingency Chi-squared test.

## Results

### *Gata2a*^*um27*^ Mutants Show Defective Primitive Myeloid Development

To define the effects of Gata2a on early developmental hematopoiesis we carried out WISH for hematopoietic markers on *gata2a^*um*27^* mutant embryos over time. The earliest hemato-vascular progenitors express Lmo2 which is required for expression of downstream erythroid and myeloid progenitors. We analyzed the expression of *lmo2* in the anterior lateral plate mesoderm (ALPM) at 10ss (14 hpf) in Gata2a^um27^ mutants to assess hematopoietic progenitors with myeloid potential. *Lmo2* expression in the ALPM was not affected by Gata2a loss indicating Gata2a is not required for anterior hemato-vascular cell specification (Supplementary Figures 2A–D). Some specification of committed early myeloid cells in the ALPM can occur independently of *lmo2* therefore we assessed early myeloid cell specification using WISH for *spi1b* and *cebpa*. At 10ss there was no difference in the expression of *spi1b* and *cebpa* in Gata2a^um27^ mutants compared to WT (Supplementary Figures 2E–L, respectively). Similarly, by 18ss (18 hpf), the expression of *spi1b* and *cebpa* show that the primitive myeloid cells have migrated and dispersed over the yolk normally in *gata2a^*um*27^* mutant embryos (Supplementary Figures 2M–O,Q–S, respectively). Quantifications show that *gata2a^*um*27^* mutant embryos have normal numbers of both *spi1b*+ and *cebpa*+ cells over the yolk (Supplementary Figures 2P,T). We also assessed primitive erythroid development in the posterior mesoderm (PM) during early development. Neither *lmo2* expression in the PM (Supplementary Figures 3A–C) nor the erythroid marker *gata1a* (Supplementary Figures 3D–F) were affected by loss of Gata2a.

**FIGURE 1:**
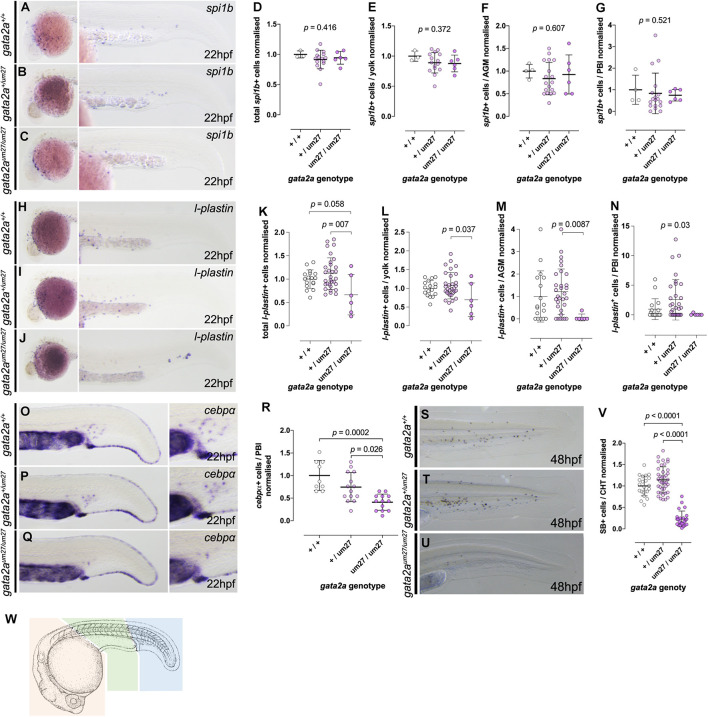
Primitive myeloid development in *gata2a* mutants at 22 hpf. **(A–C)** Expression of *spi1b* by *in situ* hybridization in 22 hpf embryos. **(A–C)** Lateral views of the head of the embryos (left) and lateral views of the tails (right), showing normal numbers of myeloid cells. **(D–G)** Quantifications of total **(D)** *spi1b*+ cells, and in the yolk **(E)**, AGM **(F)**, and the PBI **(G)**. **(H–J)** Expression of *l-plastin* by *in situ* hybridization in 22 hpf embryos. **(H–J)** Lateral views of the head of the embryos (left) and lateral views of the tails (right) show decreased *l-plastin*+cells in *gata2a*^*um27/um27*^ homozygotes. **(K–N)** Quantifications of *l-plastin*+cells in the whole embryo **(K)**, in the yolk **(L)**, AGM **(M)**, and the PBI **(N)**. **(O–Q)** Lateral views showing *cebpa* expression in the tail (left) and PBI (right) in 22 hpf embryos. **(R)** Quantification of *cebpa*+ cells in the PBI of Gata2a^*um27*^ mutants. **(S–U)** Lateral views of 48 hpf mutant embryos stained with SB, which labels granulocytes. **(V)** Quantification of SB+ cells in the CHT of 48 hpf embryos shows decreased number of granulocytes in *gata2a*^*um27/um27*^ homozygotes. **(W)** Cartoon scheme of location of cells counted in these studies. Orange region = yolk, Green = AGM, blue = PBI. Camera lucida image modified from Kimmel et al. (1995).

We next assessed primitive myeloid development in *gata2a^*um*27^* mutant embryos in both anterior and posterior mesoderm derived regions at 22 hpf. As observed at earlier stages, WISH for *spi1b* on 22 hpf *gata2a^*um*27^* mutant embryos shows normal distribution of myeloid cells both over the yolk and in the trunk and tail (Figures 1A–G) Scheme for analysis in Figure 1W. We next used WISH to assess the expression of *l-plastin*, which in some studies has been shown to specifically mark monocyte/macrophages at this stage, but is also known to be expressed in all leucocytes (Herbomel et al., 1999; Crowhurst et al., 2002). *l-plastin* expressing cells arise in the ventral mesoderm of the embryo and migrate over the yolk and trunk (Figures 1H–J, quantified in Figures 1K–N). A subset of *l-plastin* expressing cells arise in the posterior blood island (PBI) around this time, the origin of which is not clearly delineated and thought to be distinct from that of anterior derived macrophages (Herbomel et al., 1999). Quantifications on stained embryos show that the total number of *l-plastin*+ cells in markedly reduced in *gata2a^*um*27/um27^* mutant embryos is compared to that of their wildtype siblings, and significantly reduced compared to *gata2a^*um*27/+^* (Figure 1K). Further quantifications show that this difference is present across the embryo but most marked in yolk and trunk. We also investigated early myeloid lineage cells using the marker *cebpa*. Cebpa is a master regulator of granulocytic differentiation, but also plays a role in the development of eosinophils, basophils and monocytes. Similarly, we observed loss of *cebpa* expressing cells in the PBI of Gata2a mutants (Figures 1O–Q, quantified in Figure 1R). At this time point neutrophils are not yet fully differentiated, however, these cells could represent granulocyte precursors. Another possible explanation for the reduction in *cebpa* is that they may are marking early erythro-myeloid precursors (EMP) which emerge around this time, however, we did not assess EMP specifically using other markers. In summary, loss of Gata2a does not impact specification of early hematopoietic progenitors but results in loss of both anterior and posterior cells of the myeloid lineage during primitive hematopoiesis.

### Impaired Definitive Hematopoiesis in Gata2a^*um27*^ Mutants

**FIGURE 2:**
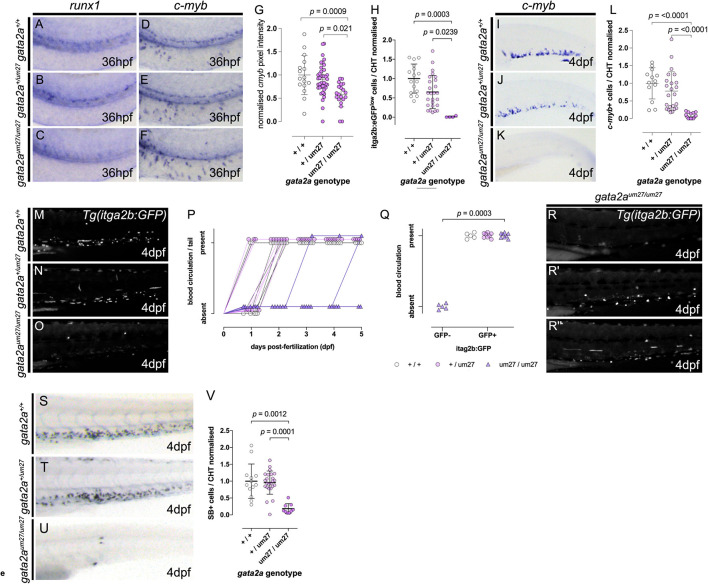
Impaired HSPC development in *gata2a* mutants. **(A–C)** Expression of *runx1* by *in situ* hybridization in 36 hpf embryos, showing decreased expression in *gata2a*^*um27/um27*^ homozygotes. **(D–F)** Lateral views of *c-myb* WISH on 36 hpf Gata2a^*um27*^ mutants showing decreased expression in in *gata2a*^*um27/um27*^ homozygotes. **(G)** Quantification of *c-myb* expression from *in situ* hybridization images in the AGM of 36 hpf embryos. In **(H)** mutant fish carrying *itga2b:GFP* transgene, labeling HSPCs that reside in the CHT, were used to quantify GFP^low^ cells in the CHT of 56 hpf embryos, showing an allele dependent decrease in GFP^*low*^ cells. **(I–K)** Lateral views of 4 dpf larvae showing the expression of *c-myb* by *in situ* hybridization. **(L)** Quantification of *c-myb*+ cells in the CHT of 4 dpf mutant larvae. **(M–O)** Lateral views of the tails of 4 dpf mutant *Tg(itga2b:GFP)* fish showing a decrease of GFP+ cells in *gata2a*^*um27/um27*^ homozygotes. In **(P)**, the presence (up) or absence (bottom) of blood flow in the tail of Gata2a^*um27*^ mutant fish was monitored each day until 5 dpf. Notice the *gata2a*^*um27/um27*^ homozygotes (purple triangles) gaining blood circulation at 3 and 5 dpf. **(Q)** Plot showing the correlation of the presence (up) or absence (bottom) of blood flow in the tail of Gata2a^*um27*^ mutant fish and the presence (right) or absence (left) of *Tg(itga2b:GFP)*+ cells in 4 dpf fish. At 4 dpf, some *gata2a*^*um27/um27*^ homozygotes (purple triangles) show GFP+ cells, all of which also exhibit circulation in the tail. **(R–R″)** Lateral views of 4 dpf mutant *Tg(itga2b:GFP)* fish showing a range of phenotypes of *gata2a*^*um27/um27*^ homozygotes. **(S–U)** Lateral views of 4 dpf mutant larvae stained with SB. **(V)** Quantification of SB+ cells in the CHT of 4 dpf larvae shows decreased number of granulocytes in *gata2a*^*um27/um27*^ homozygotes.

We next assessed definitive hematopoiesis in Gata2a mutants. HSPC numbers were assessed in Gata2a mutants using WISH for *c-myb* and *runx1*. Expression of both *runx1* and *c-myb* was reduced in Gata2a mutants (Figures 2A–F quantified in Figure 2G). This was statistically significant for *gata2a*^*um27/um27*^ only, but correlation suggested that this may be an allelic dose dependent effect (*r* = 0.88, p = 0.0002). We further quantified HSPC using epifluorescent imaging of *gata2a*^*um27*^ mutants crossed to *Tg(itga2b:GFP)* transgenic animals where static GFP^lo^ cells mark HSPC (Lin et al., 2005; Bertrand et al., 2008; Ma et al., 2011; Svoboda et al., 2014). GFP^lo^ cells appeared reduced in *gata2a*^*um27/+*^ embryos at 56 hpf, however, this did not reach statistical significance when compared to WT (Figure 2H), nonetheless, Pearson’s correlation again suggested a contributing effect across genotypes (Pearson *r* = *−*0.55, *p* = 0.0004). By contrast GFP^lo^ cells are virtually absent in *gata2a*^*um27/um27*^. We hypothesized that the near absence of stem cells in Gata2a^um27^ homozygous animals is related to its loss of trunk circulation (from discontinuous aortae or abnormal communication between the cardinal vein and aorta as shown in Supplementary Figure 4), impeding blood flow to initiate endothelial to hematopoietic transition (EHT)(Zhu et al., 2011). Migration and expansion of HSPCs was then assessed using expression of *c-myb* (Figures 2I–L) and static *Tg(itga2b:GFP)*^lo^ cells (Figures 2M–R) at 4 dpf in the caudal hematopoietic tissue (CHT, fetal liver equivalent). Again, we observed absent *c-myb* expressing HSPC in Gata2a^*um*27^ homozygous mutants (Figure 2K compared to Figures 2I,J). In this instance, there were no statistical differences between WT and heterozygous Gata2a^um27^ mutants (Figures 2M–O) suggesting that the initial production of HSPC was only delayed in *gata2a*^*um27/+*^ mutants or subsequently compensated for following migration to the CHT. During the analysis of *Tg(itga2b:GFP)* transgenic animals, we noted a proportion of *Tg(itga2b:GFP); gata2a^*um27/um27*^* embryos had GFP^lo^ cells in the CHT and circulating GFP^*hi*^ thrombocytes (Figure 2R). We hypothesi***z***ed that these escaping mutants were most likely a result of embryos where sufficient blood flow had developed in the aorta to permit EHT to occur in these animals. To definitively assess this, we directly correlated the presence and location of circulation and the presence of GFP^lo^ HSPC in the CHT in individual embryos from *gata2a*^*um27/+*^ incrosses at 4 dpf (Figures 2P,Q and Table 2). This conclusively showed that loss of Gata2a alone was insufficient to lead to complete loss of HSPC, rather the circulation defect leads to loss of HSPC migration in *gata2a*^*um27*^ mutants where blood flow is absent. Notably, the absence of tail and trunk circulation resulted in the loss of GFP expressing cells. However, these studies are insufficient to determine precisely the tissue specific role of Gata2a in HSPC development due to the vascular defect impeding further analysis.

**TABLE 2:** Blood circulation in Gata2a mutants.

Gata2a genotype	Embryo	Blood circulation			GFP^+^ cells
		Anywhere	Trunk	Tail	
+/+	b6	+	+	+	+
	c4	+	+	+	+
+/um27	b3	+	+	+	+
	b8	+	+	+	+
	b9	+	+	+	+
	c1	+	+	+	+
um27/um27	b1	+	+	+	+
	b2	+	+	−	−
	b4	+	+	+	+
	b7	−	−	−	−
	b10	−	−	−	−
	c2	+	+	+	+
	c3	+	+	+	+
	c5	+	+	−	−

We went on to analyze the effects of Gata2a loss on differentiated myeloid and erythroid cells using SB and *o*-dianisidine to label myeloid and erythroid cells, respectively. SB staining showed no significant difference between myeloid cells in heterozygous compared to WT *gata2a^*um*27^* mutants at 2 dpf, but as expected a marked reduction in myeloid cells in homozygous *gata2a^*um*27/um27^* was observed (Figures 1S–U) quantified in Figure 1V. Similarly, at 4 dpf we also observe no statistical difference between WT and *gata2a^*um*27/+^* embryos but a marked reduction in *gata2a^*um*27/um27^* (Figures 2S–V). As observed with the *Tg(itga2b:GFP)* line, a small proportion of *gata2a^*um*27/um27^* mutants show expression of myeloid cells in the CHT suggesting those embryos that escape circulatory defects and develop HSPC that are able to undergo differentiation to mature granulocytes, however, it was not possible to quantify this further in view of the vascular defects.

We also analyzed the effects of Gata2a loss on mature erythroid cells. Vascular defects can delineated in *gata2a^*um*27/um27^ o*-dianisidine stained embryos at 28 hpf (Supplementary Figures 3G–L), observed as pooling of erythroid cells in the posterior blood island secondary to the vascular defect (Supplementary Figure 3L). This highlights that erythroid cells are normally hemoglobinised in the absence of Gata2a. At 28 hpf these erythroid cells are likely to have been derived from primitive wave erythropoiesis and therefore unaffected by the defect in vascular development for initiation and differentiation.

In summary, we show that WT levels of Gata2a are essential for specifying development of stem cell numbers in the dorsal aorta, however, heterozygotes show normal HSPC in the CHT by 4 dpf. In addition, differentiation of myeloid cells is unaffected by loss of one copy of Gata2a. Biallelic loss of Gata2a shows almost complete absence of HSPCs and differentiated myeloid progeny, which can be accounted for at least in part by the absence of normal blood flow in the trunk vessels.

### The Role of Gata2b in Developmental Hematopoiesis

**FIGURE 3:**
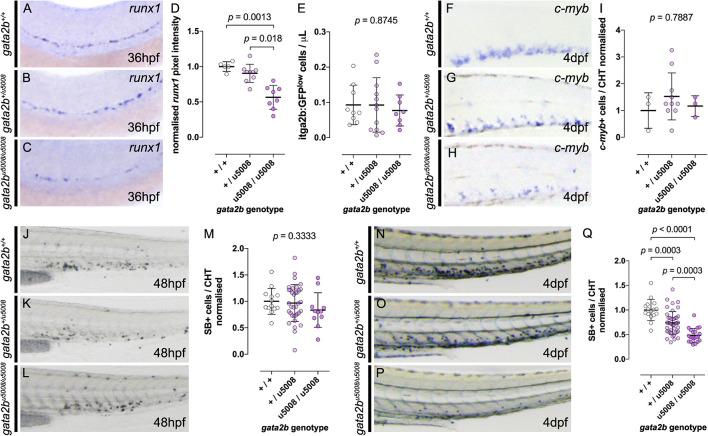
Gata2b is necessary for HSPC specification but dispensable for HSPC maturation. **(A–C)** Analysis of *runx1* expression at 36 hpf by *in situ* hybridization in Gata2b^*u5008*^ mutant embryos shows decreased expression in *gata2b*^*u5008/u5008*^ homozygotes. **(D)** Quantification of *runx1* expression in the AGM of 36 hpf mutant embryos shows allele dependent decrease of *runx1* expression. **(E)** Number of *itga2b:GFP*^lo^ cells by flow cytometry of individual *Tg(itga2b:GFP)* Gata2b^*u5008*^ mutant embryos at 3 dpf. **(F–H)** Expression of *c-myb* by *in situ* hybridization in the CHT of 4 dpf mutant larvae. **(I)** Quantification of *c-myb*+ cells in the CHT of 4 dpf mutant fish. In **(J–L)**, lateral views of 48 hpf mutant embryos stained with SB, labeling granulocytes. **(M)** Quantification of SB+ cells in the CHT of 48 hpf mutant embryos. In **(N–P)**, SB stainings of 4 dpf larvae show allele dependent decrease of SB+ cells in the CHT of Gata2b^*u5008*^ mutant fish, quantified in **(Q)**.

The *gata2b^*u*5008^* mutant was generated using CRISPR and truncates Gata2b prior to the two zinc finger domains (Supplementary Figures 1A,D). Morphological development in *gata2b^*u*5008^* mutants was normal and no defects in the vasculature or circulation were observed (Supplementary Figures 5F–K). We assessed early developmental myelopoiesis and erythropoiesis using WISH for *spi1b* and *gata1a*, respectively. We observed no defect in specification or maturation of myeloid or erythroid primitive wave hematopoietic cells in *gata2b^*u*5008^* mutants (Supplementary Figures 5A–E). We next assessed definitive hematopoiesis in *gata2b^*u*5008^* mutants. Definitive HSPC numbers in *gata2b^*u*5008^* were assessed using WISH for *runx1* at 36 hpf. We observed that loss of Gata2b led to an allele dependent reduction in *runx1* expression in the dorsal aorta (Figures 3A–C quantified in Figure 3D). This was significant only for *gata2b^*u*5008/*u*5008^*, but Pearson’s correlation was highly significant across genotypes (Pearson correlation −0.77, *p* < 0.0001). We assessed HSPC numbers further at 72 hpf using the *Tg(itga2b:GFP)* line crossed to *gata2b^*u*5008^* (Figure 3E). By this time point we saw no difference in the number of HSPC between WT and *gata2b^*u*5008^* mutants. In addition, HSPC marked by the expression of *c-myb* at 4 dpf showed no difference between genotypes (Figures 3F–H, quantified in Figure 3I). This suggests a partial requirement for Gata2b in the production of HSPC but is dispensable for HSPC maturation in the CHT where HSPC numbers recover to WT levels.

We then went on to assess the effects of Gata2b loss on myeloid differentiation using SB. At 48 hpf we observed no significant difference in myeloid cell numbers indicating that primitive and early definitive granulocytes differentiate normally (Figures 3J–L quantified in Figure 3M). By contrast at 4 dpf we observed a clear reduction in SB+ cells in *gata2b^*u*5008/+^* and *gata2b^*u*5008/u5008^* mutants in an allele dependent manner (Figures 3N–P quantified in Figure 3Q). These data show a transient reduction in definitive HSPC at 36 hpf, which recovers promptly, however, subsequent mature myeloid cell numbers are impaired with loss of Gata2b with increasing effect from loss of both alleles. This suggests compensatory mechanisms can drive normal HSPC development with loss of Gata2b, but these are insufficient to compensate for the defects in definitive myelopoiesis.

### HSPC Development in *Gata2a^+/um27^*; *Gata2b^+/u5008^* Double Heterozygotes

**FIGURE 4:**
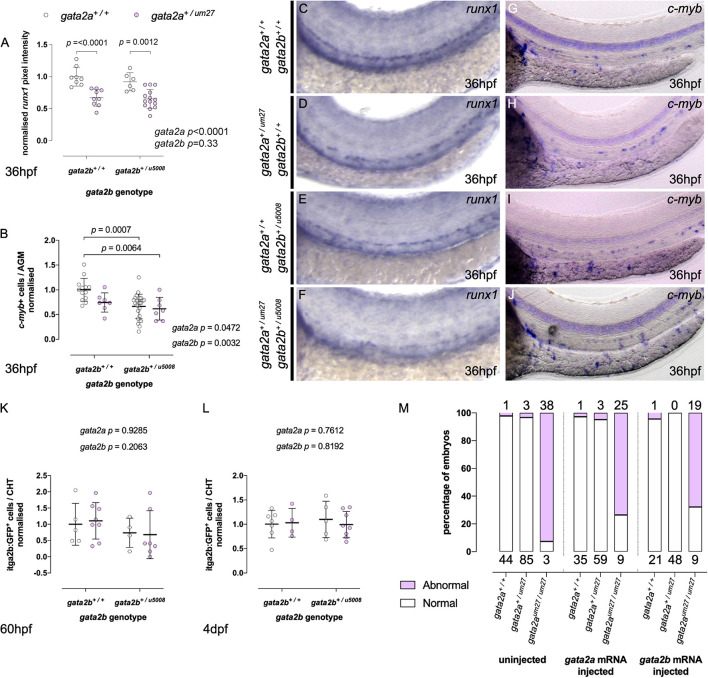
Gata2a^*um27*^ and Gata2b^*u5008*^ mutations have independent and non-additive effects on HSPC specification. **(A,B)** Quantifications of *runx1* expression **(A)** and *c-myb*+ cells **(B)** in the AGM of 36 hpf of embryos from Gata2a^+/um27^ to Gata2b^+/u5008^ crosses. **(C–J)** Expression of *runx1* **(C–F)** and *c-myb* **(G–J)** by *in situ* hybridization in 36 hpf embryos. In **(K,L)**, mutant fish carrying *itga2b:GFP* transgene, labeling HSPCs in the CHT, were used to quantify stationary GFP^lo^ cells in the CHT of 60 hpf **(K)** and 4 dpf **(L)** fish. **(M)** The percentage of embryos displaying blood flow in the trunk in embryos injected with Gata2a (middle), Gata2b (right) mRNA is compared to that of uninjected embryos (left). Absolute numbers of abnormal (pink) and normal (white) embryos are indicated above and under the columns, respectively.

We hypothesized that in order to faithfully model the effects of GATA2 deficiency seen in humans we would need to knock-out a copy of both Gata2a and Gata2b in zebrafish. To this end we crossed *gata2a^*um*27/+^* to *gata2b^*u*5008/+^* and assessed the effects on hematopoiesis. We first assessed HSPC numbers in the aorta using WISH for *runx1*. As observed in single mutant incrosses, *runx1* expression was reduced in *gata2a^*um*27/+^* and in *gata2b^*u*5008/+^* mutants (Figures 4A,C–E), with statistical significance for *gata2a^*um*27/+^* mutants. Double heterozygous mutants *gata2a^*um*27/+^*^;^*gata2b^*u*5008/+^* also showed a significant reduction in *runx1* expression compared to WT and *gata2b^*u*5008/+^* HSPC, however, there was no clear additive or synergistic effect on HSPC numbers in double heterozygotes compared to single (Figure 4F). 2-way ANOVA showed that the reduction in *runx1* expression was driven by the loss of Gata2a with no interaction between genotypes. We investigated this further using *c-myb* expression at 36 hpf. Expression of *c-myb* in the dorsal aorta was significantly reduced in both *gata2a^*um*27/+^* and *gata2b^*u*5008/+^* mutants (Figures 4B,G–J). In contrast to *runx1* expressing HSPC, 2-way ANOVA showed contribution of both alleles to the reduction in expression of *c-myb*, however, no interaction between genotypes was observed with no significant change in *c-myb* expression between single and double heterozygotes. These data suggest that Gata2a and Gata2b are both required for normal HSPC production, and that compound heterozygosity is not additive or synergistic in this effect. This suggests that the WT allele of either Gata2 gene can compensate for the other in HSPC. The difference between effects observed with *runx1* and *c-myb* may reflect a more myeloid biased HSPC in the *c-myb* expressing cells, since *c-myb* expression is greater in myeloid committed HSPC and Gata2b appears to have a role in definitive myeloid development. Alternatively, *c-myb* expressing cells may be highlighting a more proliferative subset of HSPC (Zhang et al., 2011).

We also assessed HSPC migration and expansion by analyzing *Tg(itga2b:GFP)^lo^* cells in the CHT at 60 hpf and 4 dpf. As observed in single heterozygotes, HSPC numbers recover and show no difference between genotypes at this timepoint (Figures 4K,L).

To further delineate the ability of Gata2b to rescue the effects of Gata2a we injected Gata2b mRNA into *gata2a^*um*27/+^* incross animals. We used blood flow through the trunk as a readout of rescue in *gata2a^*um*27^* mutants. In uninjected mutants we observed 7% of *gata2a^*um*27/um27^* embryos with bloodflow through the trunk region by 3 dpf in keeping with their known vascular defects. Expression of Gata2a mRNA rescued this to 26% and Gata2b mRNA to 32% with blood flow through the trunk indicating Gata2b is as effective at rescue of the vascular defects induced by Gata2a loss as Gata2a (Figure 4M).

### *Gata2a^+/um27^*; *Gata2b^+/u5008^* Double Heterozygotes Show Distinct Defects in Primitive and Definitive Myeloid Development

**FIGURE 5:**
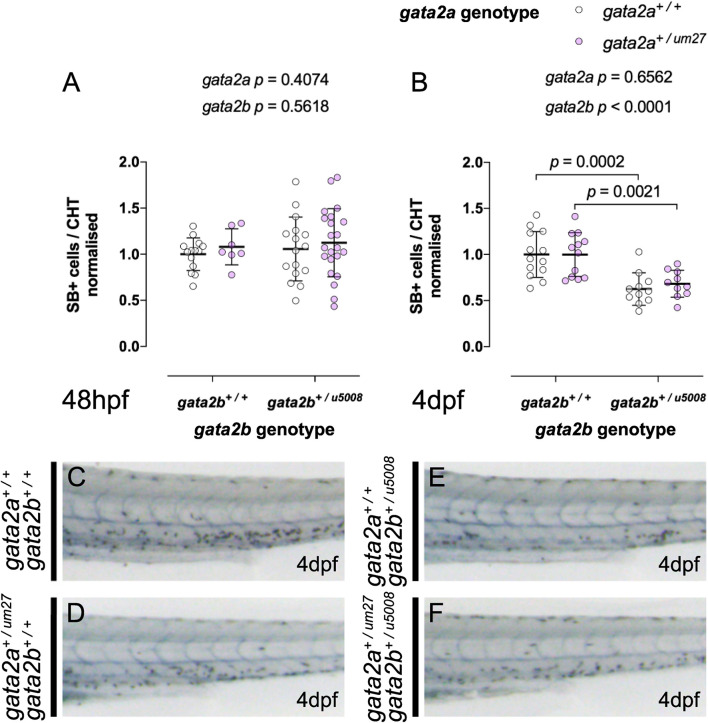
Gata2b^+/u5008^ mutants display impaired definitive granulopoiesis independent of *gata2a* genotype. **(A,B)** Quantifications of SB+ cells in the CHT of 48 hpf **(A)** and 4 dpf **(B)** fish from a Gata2a^+/um27^ to Gata2b^+/u5008^ cross. **(C–F)** Lateral views of the trunk of SB stained larvae at 4 dpf showing decreased SB+ cells in the CHT of Gata2b^+/u5008^ fish.

We next went on to assess the effect of *gata2a^+/um27^; gata2b^+/u5008^* double heterozygosity on myeloid development since we had observed myeloid defects in both *gata2a^*um*27^* (in primitive myelopoiesis) as well as in *gata2b^*u*5008^* (in definitive hematopoiesis). Using SB as a readout, we did not observe any difference between the number of granulocytes in *gata2a^+/um27^; gata2b^+/u5008^* double heterozygotes compared to single heterozygotes or WT at 2 dpf (Figure 5A). At 4 dpf we observed a significant reduction in SB+ granulocytes in *gata2b^*u*5008/+^* but no additional effect with the loss of *gata2a^*um*27^* (Figures 5B–F). These findings confirm that Gata2a heterozygosity does not contribute to the development of SB+ granulocytes alone or in combination with Gata2b loss, and that loss of a single allele of Gata2b is sufficient to confer defective definitive developmental myelopoiesis.

### Effects of *Gata2a^+/um27^* and *Gata2b^+/u5008^* on Adult Hematopoiesis

Our underlying hypothesis is that combined heterozygous loss of gata2a and gata2b will model human GATA2 mediated diseases. To assess the effects of single and combined heterozygous mutants in adults we undertook flow cytometric analysis of whole kidney marrow (WKM) at 12 and 15 months post of age. Blood populations were assess based on forward and side scatter characteristics, with the addition of back-gating GFP and mCherry expressing populations (Supplementary Figures 6A,B). No changes in populations were observed at 12 months, however, by 15 months we observed a significant reduction in the myeloid compartment in *gata2b^*u*5008/+^* and *gata2a^*um*27/+^;gata2b^*u*5008/+^*. On more detailed analysis of the myeloid compartment we assessed the proportion of cells within the myeloid gate that expressed the mCherry transgene. Double heterozygous *gata2a^*um*27/+;^gata2b^*u*5008/+^* animals showed a statically significant reduction in the expression of mCherry at both 12 and 15 months of age. The lysozyme C promoter has been shown in zebrafish models to drive expression in myelomonocytic cells including a proportion subset of neutrophils (Hall et al., 2007). Therefore we think that the reduction in expression of mCherry may represent either reduced numbers, or reduced function of neutrophils or other myelomonocytes in double heterozygotes. This is in keeping with patients who deficient in GATA2 who may show phenotypes of monocytopenia, or abnormal granulopoiesis related to MDS.

We did not, however, observe any leukemias in our double heterozygous mutants, or any overt features of defective lymphatic function as observed in Embergers syndrome. Our phenotype is notably more subtle that that observed in gata2ai^4/i4^ (Dobrzycki et al., 2020). We think this is likely because the gata2a^*i*4^ phenotype is a tissue specific knockdown of Gata2a in hematopoietic cells and occurs in homozygous gata2ai^4/i4^, therefore the level of Gata2a is likely significantly lower than in our mutants that are not viable as homozygotes. Also gata2a i4 mutants have reduced levels of Gata2b, potentially adding additional impact to their phenotype. Thus our data supports the fact that double heterozygous mutants exhibit features of GATA2 deficiency, but that there may be compensatory mechanisms from the other ohnolog that ameliorate some of the phenotypes.

## Discussion

We sought to develop a model of human GATA2 haploinsufficiency using zebrafish to further understand the etiology and spectrum of disease related to effects on developmental hematopoiesis. To do this we proposed analysis of loss of Gata2a and Gata2b alone and in combination in zebrafish would recapitulate a range of phenotypes that may contribute to disease evolution and may further infer structure-function relationships inherent from the sequence similarities and differences between these two highly similar ohnologs. In addition, we hypothesized that we may uncover important redundancies between these and potentially other GATA factors.

We showed that both Gata2 genes are syntenic with human chromosome 3, indicating that these genes arose from an ancestral additional round of duplication of this region. The expression pattern of Gata2a and Gata2b genes are disparate with limited overlap during development (Butko et al., 2015).

Our studies show that loss of Gata2a or Gata2b result in a reduction of HSPC. Prior studies have shown that *gata2a^*um*27/um27^* mutants have severe vascular defects (Zhu et al., 2011) which are likely to elicit hematopoietic phenotypes as a secondary consequence. However, our data suggest that *gata2a^*um*27/um27^* homozygotes are capable of recovering and developing HSPCs in a few cases where blood flow was recovered or truncal vasculature remained intact. These observations are consistent with the recovery of HSPC development reported in fish with mutations in a conserved enhancer that drives endothelial expression of Gata2a (Dobrzycki et al., 2020). Other recent studies have shown that initial specification and endothelial-to-hematopoietic transition of HSC is intact in Gata2b knockout animals (Gioacchino et al., 2021). In our studies we demonstrated that *Gata2b^*u*5008/u5008^* mutants have normal vascular development but have a reduction in HSPCs in the aorta from 36 hpf. This defect is no longer evident by 4 dpf in the CHT and notably overall their phenotype is milder than that observed in *gata2b* morphants (Butko et al., 2015). Interestingly, analysis of HSPC development in compound heterozygotes shows slightly more severe phenotypes on *gata2a^*um*27/+^* heterozygotes, consistently with the role of Gata2a upstream of Gata2b, regulating *runx1* and *gata2b* expression in the hemogenic endothelium (Dobrzycki et al., 2020).

Analysis of *gata2a^*um*27^* mutants shows a role for Gata2a during primitive myelopoiesis while analysis of *gata2b^*u*5008^* mutants show that Gata2b is dispensable for primitive myelopoiesis but is required for definitive myelopoiesis. This phenotype can be observed at 4 dpf and is consistent with decreased myeloid cells in the whole kidney marrow of adult Gata2b mutants (Gioacchino et al., 2019). Butko et al. (2015) showed using an elegant genetic switch experiment and *gata2b* derived promoter elements that the majority of adult myeloid cells are derived from *gata2b*+ cells. We propose that the role for Gata2b in myelopoiesis is independent from HSPC production, as experiments on heterozygote *gata2b^+/u5008^* fish show normal HSPC numbers as soon as 60 hpf. Additionally, *Gata2b^*u*5008^* mutants exhibit normal definitive erythropoiesis, suggesting that Gata2b is required specifically for granulocyte development. This is consistent with findings of neutropenia in patients carrying *GATA2* mutations (Pasquet et al., 2013; Wlodarski et al., 2016).

Our results show that both Gata2a and Gata2b are required for myeloid and HSPC development, each at different developmental stages. A possible explanation could be the sub-functionalization of these Gata2 ohnologs, by exclusion of territories of expression. Expression analysis shows that *gata2a* is expressed as early as 8ss in the PLM, while *gata2b* expression is much more spatially restricted and can only be detected from 18ss (Butko et al., 2015). Consistently, we found successive requirement for Gata2a and Gata2b for myeloid and HSPC development. We show that there is an early requirement for Gata2a in primitive myelopoiesis in the PBI, and a later requirement for Gata2b in definitive granulopoiesis in the CHT. Similarly, while Gata2a is necessary for vascular development, Gata2b is required later during HSPC emergence.

The differing contributions of Gata2a and Gata2b could also be explained by expression exclusion without substantial changes in protein function. The observed non-synergistic effects on HSPC development in compound heterozygotes suggest that over time, one WT copy of each Gata2 gene is sufficient to compensate the lack of the other. This supports the possibility that the differing phenotypes are a product of expression exclusion rather than different protein function. Further support to this comes from circulation rescue experiments which show that Gata2b overexpression is able to partially rescue blood flow in the trunk of Gata2a^um27/um27^ homozygotes to a similar level as Gata2a. Rescue studies in Gata2b morphants conversely could not be rescued by constitutive expression of Gata2a (Butko et al., 2015), although notably GATA factor redundancy between other GATA genes has also been described. Therefore compensatory mechanisms from other GATA factors already is an alternative explanation (Ferreira et al., 2007).

Adult *gata2a^*um*27/+;^gata2b^*u*5008/+^* animals show defects in the myeloid compartment which we first observe at 12 months of age consistent with features of GATA2 deficiency. However, the range of phenotypes and rate if onset is less florid than an alternative published zebrafish Gata2 disease model (Dobrzycki et al., 2020), where a tissue specific intronic enhancer regulating Gata2a expression is completely removed (and downstream of this Gata2b). This further suggests a dose dependent effect of Gata2a in hematopoietic cells may drive the leukemic phenotype, or the acquisition of second hit mutations that drive the formation of leukemia’s in GATA2 deficiency.

In summary, we describe the sequential and complementary defects resulting from loss of Gata2a and Gata2b in zebrafish during development and in adults, further expanding our understanding of the complex inter-regulation of these GATA factors during hematopoiesis, and their role in developmental hematopoiesis relevant to human GATA2 deficiency.

## Data Availability Statement

The original contributions presented in the study are included in the article/Supplementary Material, further inquiries can be directed to the corresponding author/s.

## Ethics Statement

The animal study was reviewed and approved by the UK Home Office.

## Author Contributions

OP designed and performed the experiments, interpreted data, wrote, and edited the manuscript. AL, JR, YH, NK, and HL designed, performed the experiments, and interpreted data. CM analyzed and interpreted the experiments. CU performed the experiments and interpreted data. EP conceived the study, directed the experimental design, provided resources, and wrote the manuscript. All authors contributed to the article and approved the submitted version.

## Conflict of Interest

The authors declare that the research was conducted in the absence of any commercial or financial relationships that could be construed as a potential conflict of interest.

## Publisher’s Note

All claims expressed in this article are solely those of the authors and do not necessarily represent those of their affiliated organizations, or those of the publisher, the editors and the reviewers. Any product that may be evaluated in this article, or claim that may be made by its manufacturer, is not guaranteed or endorsed by the publisher.

## Footnotes

1
http://chopchop.cbu.uib.no/
