# Supplementary material for: Differential Requirement of Gata2a and Gata2b for Primitive and Definitive Myeloid Development in Zebrafish
Source: Front Cell Dev Biol. 2021 Sep 13;9:708113. doi: 10.3389/fcell.2021.708113 (PMC8475954; doi:10.3389/fcell.2021.708113)
Supplement: Supplementary file 1 [file Data_Sheet_1.PDF]

Supplementary Figures and legends

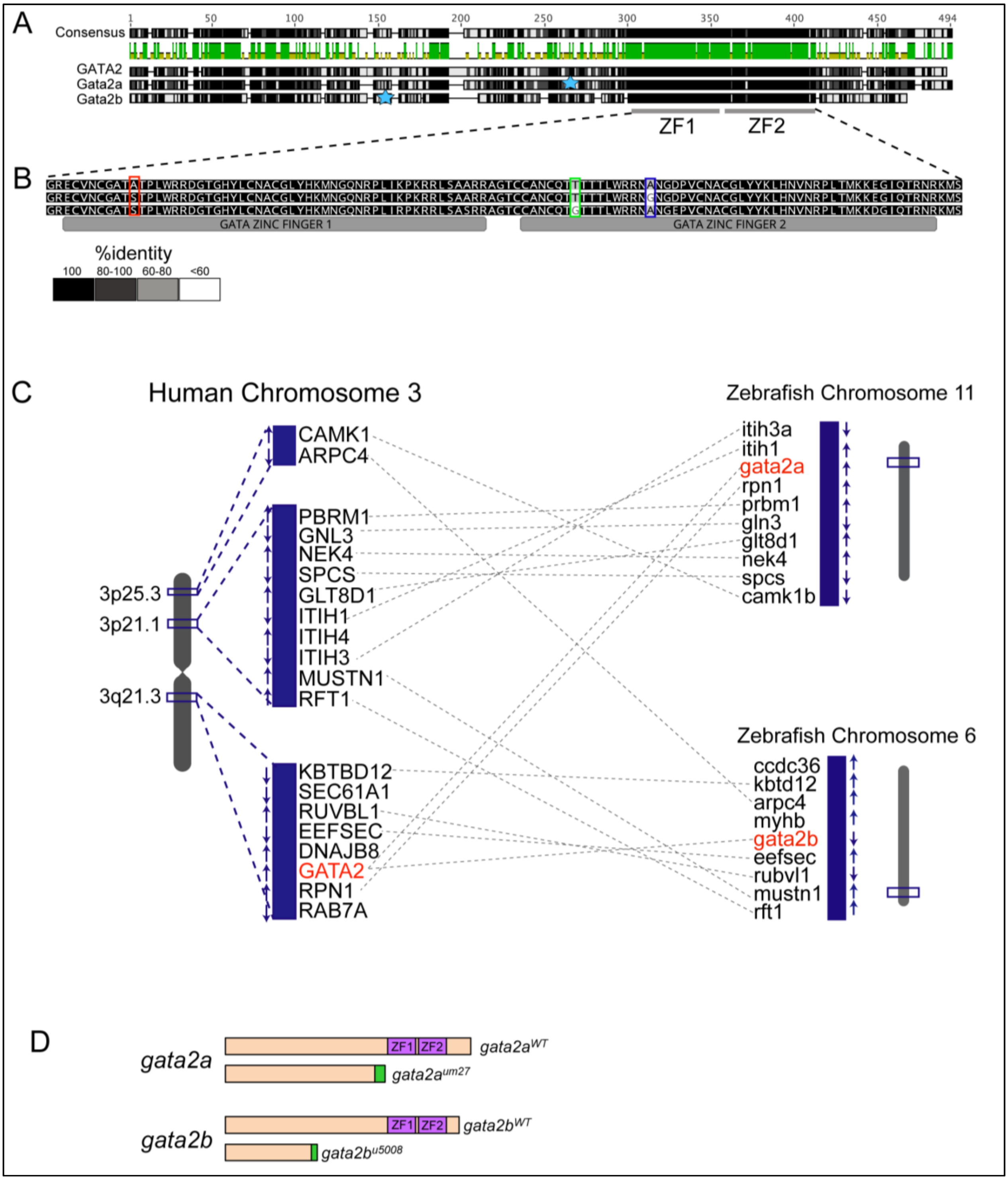

Supplementary Figure S1. Zebrafish Gata2 ohnologs result from whole genome duplication and are syntenic with human GATA2. (A-B) alignment of human GATA2 and zebrafish Gata2a and Gata2b protein sequences. Blue starts indicate the location of the mutations in Gata2a^{um27} and Gata2b^{u5008} used in this study (B) Identities in the zinc finger region show only 3 regions where human and zebrafish genes differ highlighted by coloured boxes. Notably the threonine residue corresponding to green box is within a group of 5 threonine residues that are sites of recurrent mutations or deletions associated with leukaemia predisposition. This residue is changed to Glycine in Gata2b (C) Gene-level syntenic relationships between human *GATA2* and zebrafish *gata2a* and

gata2b. (D) Schematic of the truncated proteins for Gata2a and Gata2b produced in Gata2a^{um27} and Gata2b^{u5008} alleles. Green region depicts frameshifted sequence prior to premature stop codon.

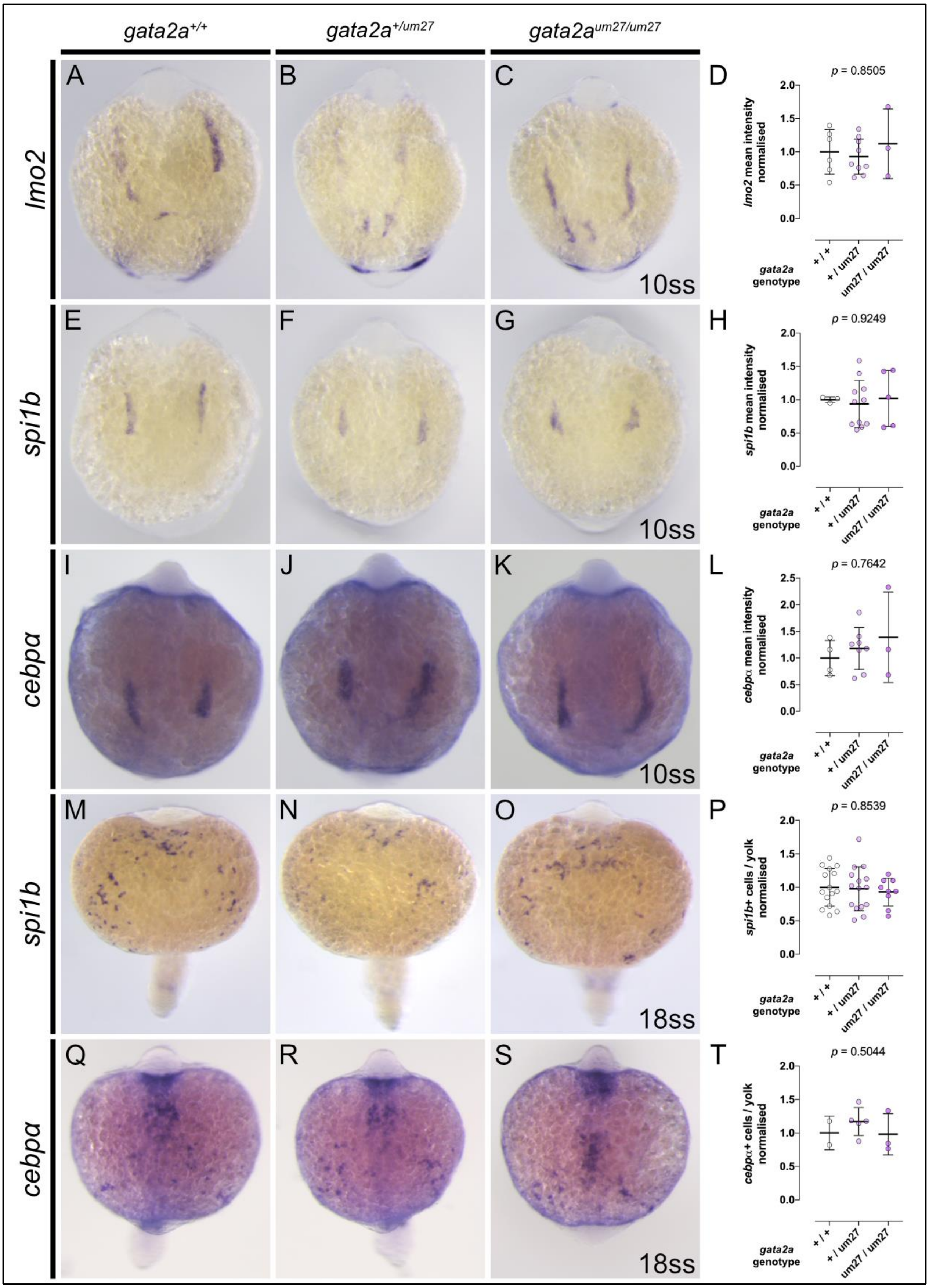

Supplementary Figure S2. Gata2a^{um27} mutants show normal primitive haematopoiesis. (A-C) Anterior views of 10ss Gata2aum27 mutants showing *lmo2* expression. (D) Quantification of *lmo2* expression in the ALPM suggest normal specification of haemato-vascular progenitors. (E-G) Analysis of *spi1b* expression by *in situ* hybridization in 10ss Gata2a^{um27} mutants. (H) Quantification

of *spi1b* expression showing normal myeloid development. (I-K) WISH images showing normal *cebpa* expression at 10ss in Gata2a^{um27} mutants, quantified in (L). (M-O) Anterior views of 18ss Gata2a^{um27} mutants showing the expression of *spi1b* in primitive myeloid cells on the yolk. Quantification in (P) shows Gata2a^{um27} mutants display normal numbers of *spi1b+* cells. (Q-S) Expression of *cebpa* in 18ss Gata2a^{um27} mutants. (T) Quantification of *cebpa+* cells shows no difference across genotypes. All images show dorsoanterior views with anterior to the bottom, and dorsomedial portion of the embryo to the top. (A-L) 10ss embryos, and (M-T) 18ss.

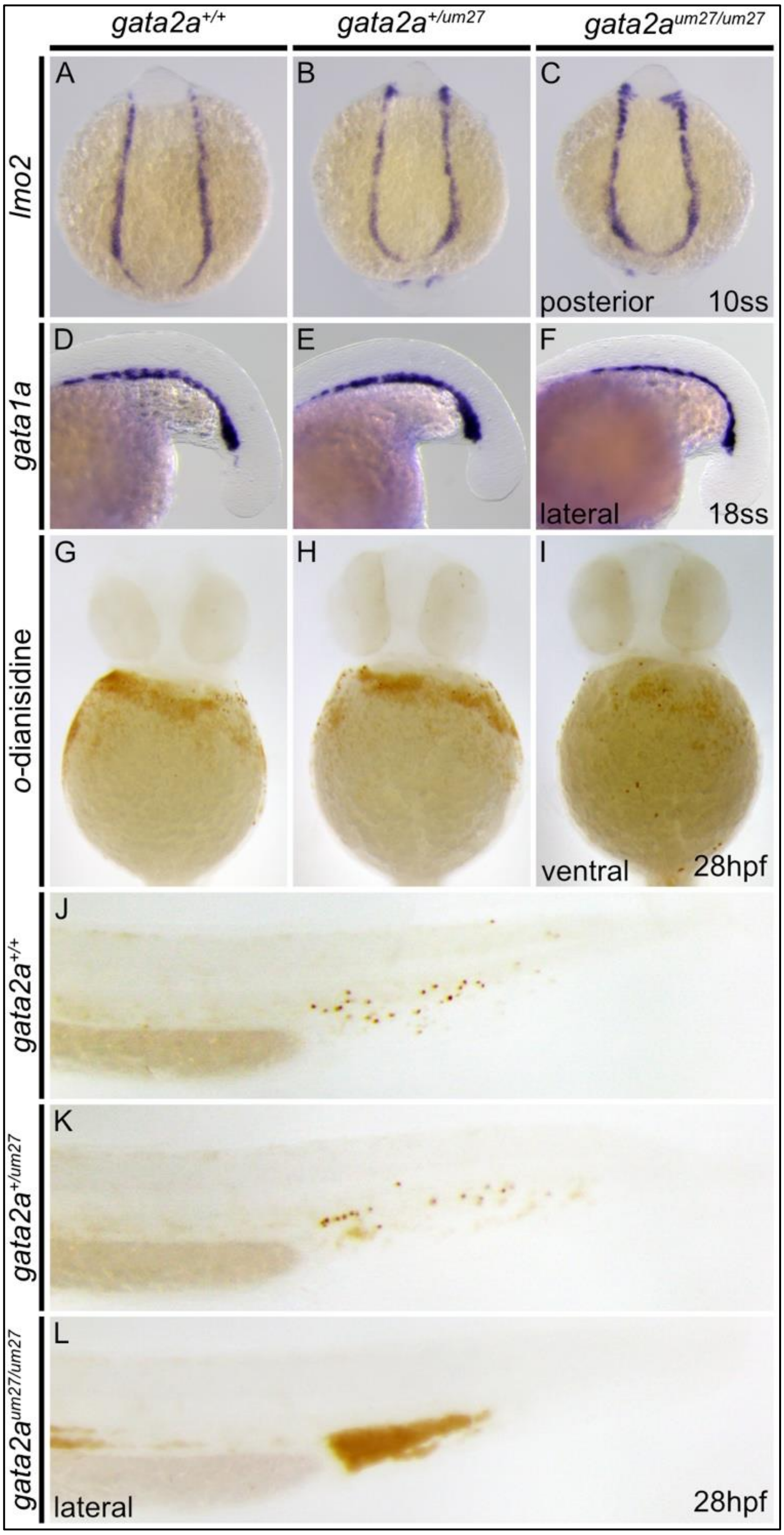

Supplementary Figure S3. Normal erythroid development in Gata2a^{um27} mutants. (A-C) Posterior views of *lmo2* expression analised by WISH on 10ss Gata2a^{um27} mutants shows normal expression on the PM. (D-F) Lateral views of 18ss Gata2a^{um27} mutants showing the expression of the erythroid marker *gata1a*. (G-L) Images of *o*-dianisidine stainings on 28hpf Gata2a^{um27} mutants. (G-I) Ventral views of the head of Gata2a^{um27} mutants showing erythroid cells over the yolk. (J-L) Lateral

views of the tails of Gata2a^{um27} mutants. Notice the decrease relative in erythroid cells over the yolk of *gata2a$^{um27/um27}$* homozygote in (I), and the pooling of erythroid cells in the tail (L, arrowhead).

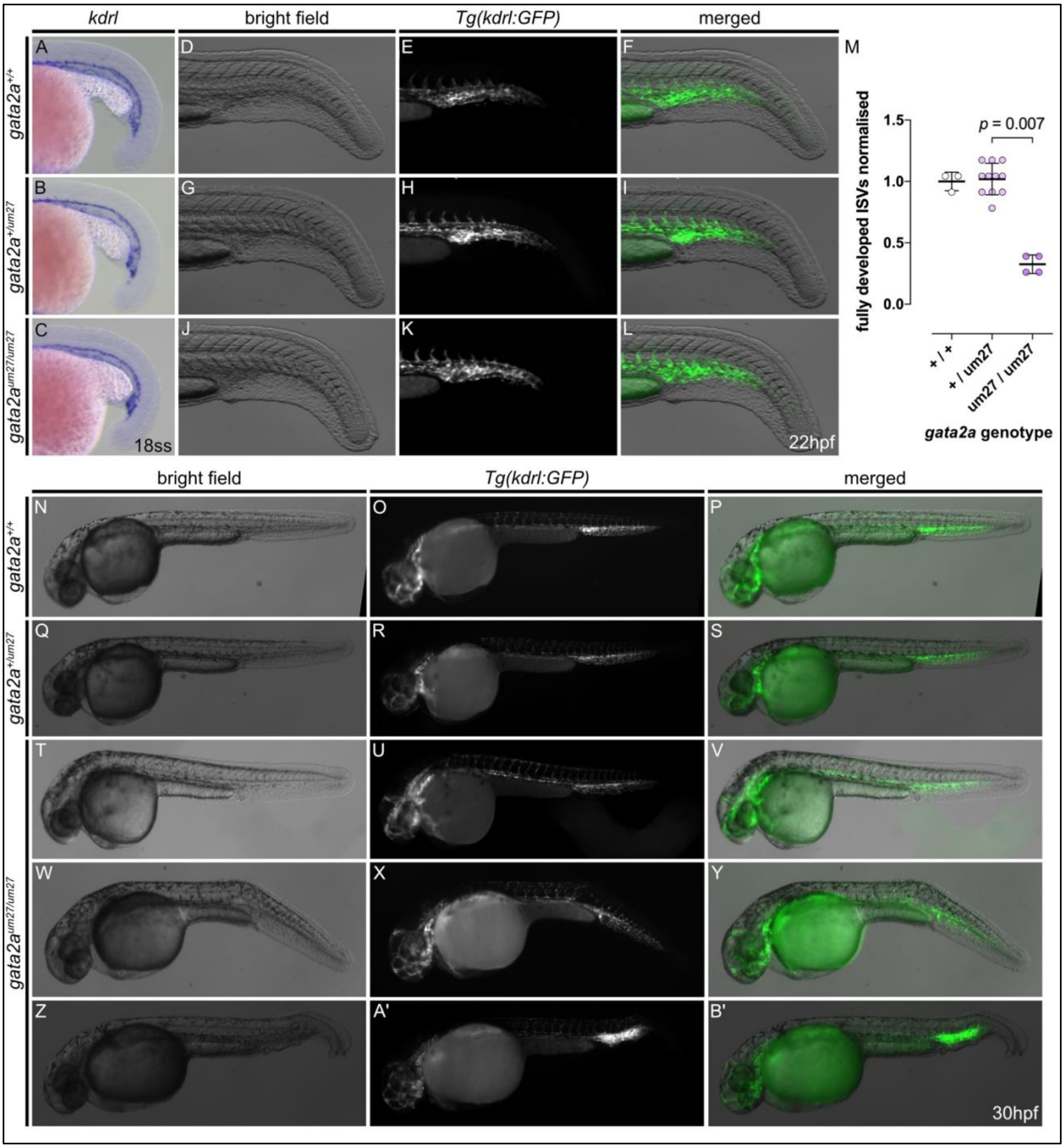

Supplementary Figure S4. Gata2a loss induces diverse vascular phenotypes. (A-C) Lateral views of 18ss Gata2a^{um27} mutants showing the expression of vascular marker *kdrl*. (D-L) Lateral views of Gata2a^{um27} mutants crossed to *Tg(kdrl:GFP)* transgenic animals, where vasculature expresses GFP. Dorsal elongation of ISV is delayed in *gata2a$^{um27/um27}$* homozygotes. (M) Quantification of fully developed ISV in 22hpf Gata2a^{um27} mutants. (N-B') Lateral views of Gata2a^{um27} mutants crossed to *Tg(kdrl:GFP).* Notice normal vascular development in heterozygote (Q-S) compared to WT embryo (N-P). In contrast, *gata2a$^{um27/um27}$* homozygotes (T-B') exhibit

diverse phenotypes, ranging from a mild phenotype with discontinuous aorta (T-V), to antero-posterior axis defects (W-Y), and severe vascular defects with extended anastomosis (Z-B').

ISV = intersegmental vessel.

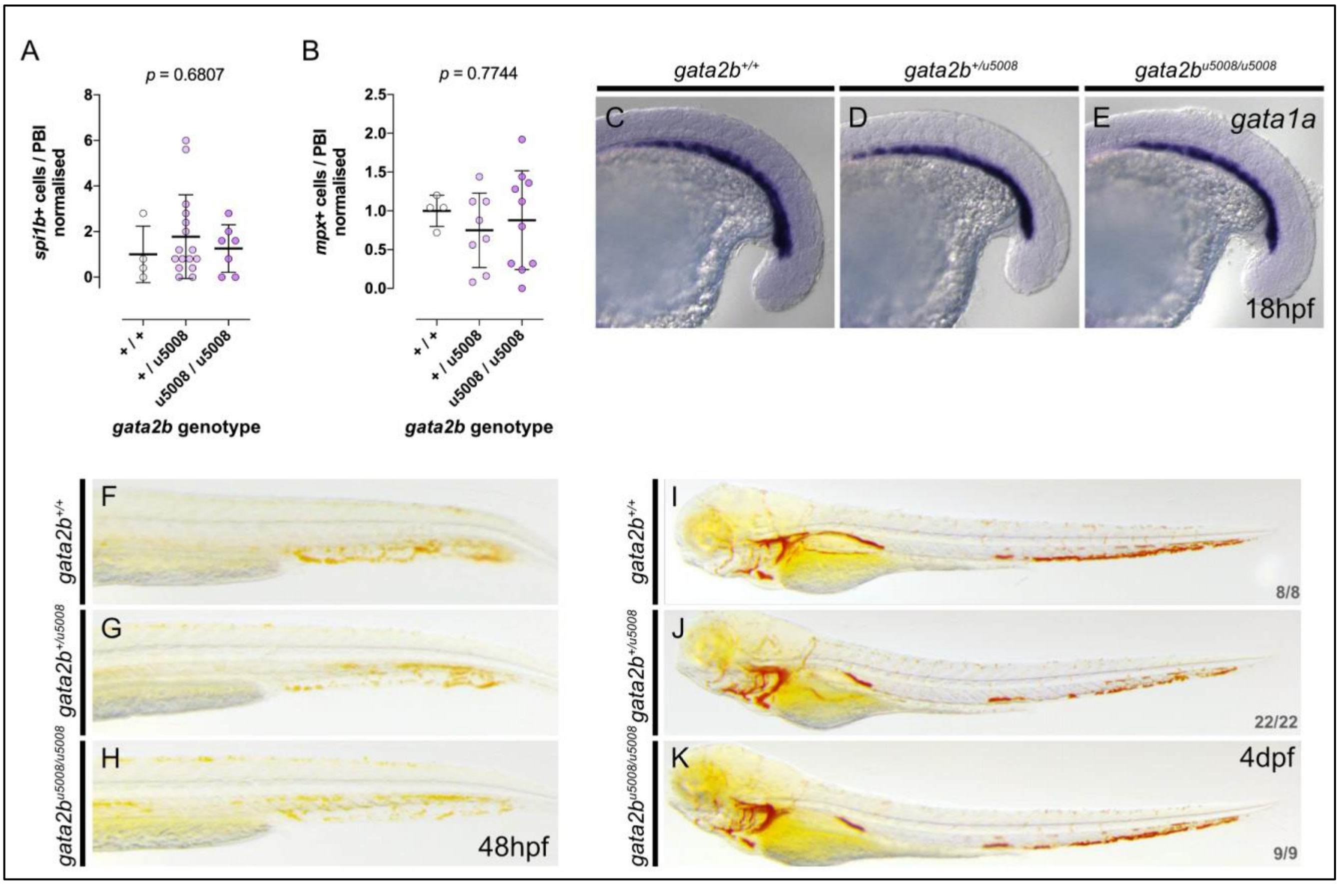

Supplementary Figure S5. Erythroid and primitive myeloid development in Gata2b^{u5008} mutants. (A-B) Myeloid development was analysed by WISH for myeloid markers *spi1b* (A) and *mpx* (B) on 22hpf Gata2b^{u5008} mutants. (A) Quantification of *spi1b+* cells on the PBI of Gata2b^{u5008} mutants. (B) Quantification of *mpx+* cells on the PBI of Gata2b^{u5008} mutants. (C-E) Lateral views of 18hpf Gata2b^{u5008} mutants showing the expression of erythroid marker *gata1a.* (F-K) Lateral views of *o*-dianisidine stainings showing normal haemoglobinisation on Gata2b^{u5008} mutants at 48hpf (F-H) and 4dpf (I-K).

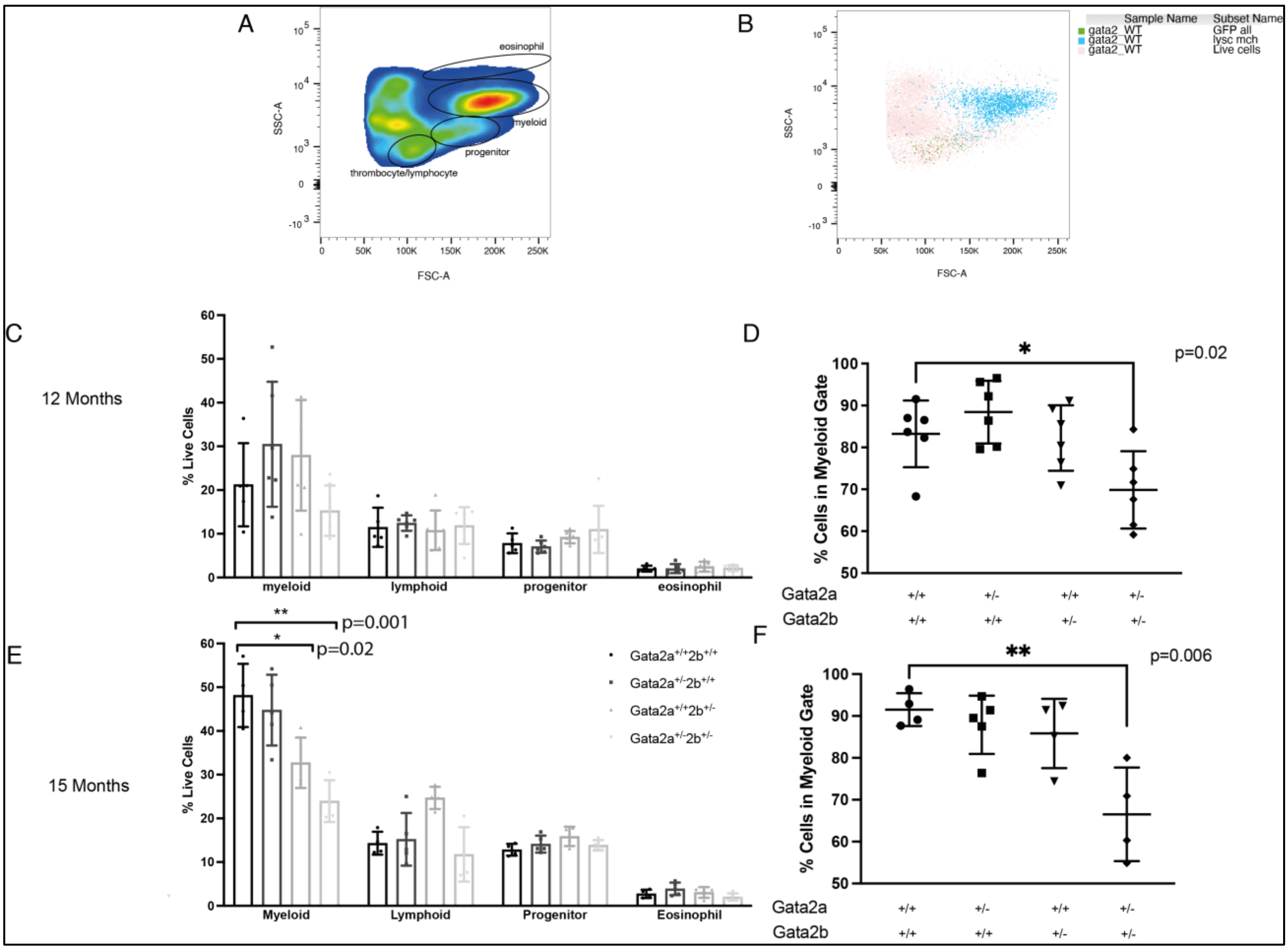

Supplementary Figure S6 - Adult Gata2 mutants show myeloid defects

Flow cytometric analysis of adult Gata2a$^{um27/-}$ and Gata2b$^{u5008/-}$ single and double mutants Mutants carry the Tg(lyzC:mCherry) and Tg(itga2b:eGFP) transgenes.

(A) Scheme of forward scatter vs side scatter plots defining gates used for analysis. (B) Validation of gates by back-gating using mCherry expression (myeloid gate) and eGFP expression for progenitor (C, E) phenotype of cells by FSC x SSC gating at 12 months and 15 months respectively.

FIGURE 1	replicate experiments
1D	2
1E	2
1F	2
1G	2
1K	3
1L	3
1M	3
1N	3
1R	4
1V	3

FIGURE 2	
2G	2
2H	2
2L	1
2P/Q	1
2V	3

FIGURE 3	
3D	2
3E	1
3I	2
3M	2
3Q	2

Supplementary Table 1: experimental replicates

FIGURE 4	
4A	2
4B	2
4K	2
4L	1
4M - uninjected	3
4M - gata2a mRNA injected	3
4M - gata2b mRNA injected	3

FIGURE 5	
5A	3
5B	2
